# Increased room temperature ferromagnetism in Co-doped tetrahedral perovskite niobates

**DOI:** 10.1098/rsos.210121

**Published:** 2021-10-06

**Authors:** Yi Zhou, Qing He, Fei Zhou, Xingqi Liao, Yong Liu, Zhonghong Lai, Mingqing Liao, Tianyi Han, Yudong Huang, Jingchuan Zhu

**Affiliations:** ^1^ School of Materials Science and Engineering, Harbin Institute of Technology, 150001 Harbin, People's Republic of China; ^2^ MIIT Key Laboratory of Critical Materials Technology for New Energy Conversion and Storage, School of Chemistry and Chemical Engineering, Harbin Institute of Technology, 150001 Harbin, People's Republic of China; ^3^ National Key Laboratory for Precision Hot Processing of Metals, Harbin Institute of Technology, 150001 Harbin, People's Republic of China; ^4^ Center of Analysis and Measurement, Harbin Institute of Technology, 150001 Harbin, People's Republic of China; ^5^ National Key Laboratory of Science and Technology on Advanced Composites in Special Environments, Harbin Institute of Technology, 150001 Harbin, People's Republic of China

**Keywords:** room temperature ferromagnetism, perovskite niobates, BaNbO_3_

## Abstract

Dilute magnetic semiconductors (DMSs), such as (In, Mn)As and (Ga, Mn)As prototypes, are limited to III–V semiconductors with Curie temperatures (*T*_c_) far from room temperature, thereby hindering their wide application. Here, one kind of DMS based on perovskite niobates is reported. BaM*_x_*Nb_(1−*x*)_O_3−*δ*_ (*M* = Fe, Co) powders are prepared by the composite-hydroxide-mediated method. The addition of *M* elements endows BaM*_x_*Nb_(1−*x*)_O_3−*δ*_ with local ferromagnetism. The tetragonal BaCo*_x_*Nb_(1−*x*)_O_3−*δ*_ nanocrystals can be obtained by Co doping, which shows strong saturation magnetization (*M*_sat_) of 2.22 emu g^−1^, a remnant magnetization (*M*_r_) of 0.084 emu g^−1^ and a small coercive field (*H*_c_) of 167.02 Oe at room temperature. The *ab initio* calculations indicate that Co doping could lead to a 64% local spin polarization at the Fermi level (*E*_F_) with net spin DOS of 0.89 electrons eV^−1^, this result shows the possibility of maintaining strong ferromagnetism at room temperature. In addition, the trade-off effect between the defect band absorption and ferromagnetic properties of BaM*_x_*Nb_(1−*x*)_O_3−*δ*_ is verified experimentally and theoretically.

## 1. Introduction

Since the discovery of ferromagnetism in Mn-doped InAs in 1992 [1], dilute magnetic semiconductors (DMSs) with doped transition metal elements have been increasingly studied due to their fascinating multifunctional spintronic properties [2–4]. However, the solubility of doped transition metal atoms in a matrix remains a technical challenge, limiting these materials to the preparation of epitaxial films with metastable phase structures. Furthermore, elevating the *T_c_* of DMSs to room temperature is a long-standing request for their industrial application, and the complex carrier doping strategy can increase the *T_c_* of traditional DMSs to 100–180 K [5,6]. However, reports show that developing DMSs with transition metal oxides, such as Zn_1−*x*_Mn*_x_*O_2_ and Ti_1−*x*_Co*_x_*O2, can extend the critical temperature above 300 K while maintaining a relatively low *M*_sat_ ferromagnetism value of approximately 10^−2^ emu g^−1^ [7,8].

Efforts to develop the room temperature DMSs in the form of powders or bulk ceramics have been carried out on Mn-doped ZnO; however, the formation of Mn clusters under high processing temperature (*T* > 700°C) would lead to suppression or disappearance in ferromagnetism [7]. Hence, the exploration of methods with lower synthesis temperature is of great importance to the industry application of DMSs in the bulk device and, extend the room temperature DMSs to other oxides system.

Perovskite niobates, with the composition of ANbO_3_ (A = Li, K, Na, Ag), are widely used in lead-free piezoelectric and nonlinear optical devices due to their excellent ferroelectricity and nonlinear optical properties [9–14]. Previous theoretical and experimental work has proven that local ferromagnetism can be obtained in transition-metal-doped perovskite niobates [15–17]. Specifically, as a prototype of an A-site atom with an oxidation state of +2, BaNbO_3_ has shown great potential as a room temperature DMS, showing that the introduction of oxygen defects, modulation of the electric field and Co doping with transition metals can be achieved; however, its *M*_sat_ is limited to approximately 10^−2^ emu g^−1^ [18–20].

## 2. Material and methods

### 2.1. Experimental procedure

#### 2.1.1. Synthesis of *M*-doped BaNbO_3−*δ*_

*M*-doped BaNbO_3−*δ*_ nanocrystals were prepared by the composite-hydroxide-mediated method. In a typical synthesis, 3.8819 g of NaOH, 5.1181 g of KOH, 0.2660 g of Nb_2_O_5_ and 0.4880 g of BaCl_2_ · 2H_2_O were weighed and mixed, and then 0.2705 g of FeCl_3_ · 6H_2_O, or 0.2379 g of CoCl_2_ · 6H_2_O was added into, respectively. The mixture was stirred and put in a 40 ml Teflon beaker. The Teflon beaker was placed in a preheated furnace at 195°C for 24 h. Then the Teflon beaker was taken out for natural cooling to room temperature. The reaction mixture was washed and filtered by distilled water and alcohol alternately three times. The filtered *M*-doped BaNbO_3−*δ*_ powder was dried at 60°C for 4 h.

#### 2.1.2. Sample characterization

XRD was performed on a PANalytical Empyrean instrument outfitted with a PIXcel 2D detector operating at 40 kV per 40 mA, using Cu–Kα radiation (*λ* = 1.5405 Å). A Quanta 200FEG field emission SEM with EDS attachment was used for SEM analysis. XPS data were collected by ESCALAB 250Xi photoelectron spectrometer, which is produced by ThermoFisher company; the gun source was Al–Kα radiation. The HRTEM and SAED experiment was using a Tecnai G2 F30 transmission electron microscope. A Lake Shore 7404 vibrating sample magnetometer, with an external magnetic field sweeping from −15 000 to +15 000 Oe, was employed to evaluate the ferromagnetism of *M*-doped BaNbO_3−*δ*_ nanocrystals.

### 2.2. Theoretical calculation

DFT calculation in this study was performed with the Cambridge Serial Total Energy Package (CASTEP). A 2 × 2 × 2 supercell crystal model of pure BaNbO_3_ was established first. Then one of the Nb atoms in the crystal was replaced by *M* elements, accompanied by one O vacancy in the oxygen octahedral cage. Localized density approximation (LDA) was employed for geometry optimization and property calculations with CA-PZ exchange-correlation functional. The plane-wave cut-off energy was set to 400 eV, and the *k*-point sampling grid was 4 × 4 × 4.

## 3. Results

**Figure 1.**
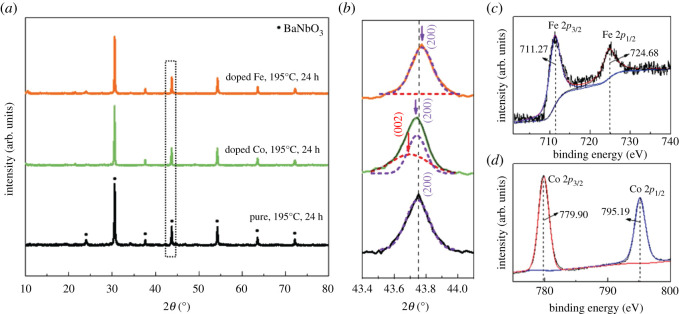
Crystal structure and elemental analysis of BaM*_x_*Nb_(1−*x*)_O_3−*δ*_. (*a*) XRD patterns of the BaM*_x_*Nb_(1−*x*)_O_3−*δ*_ nanocrystals. (*b*) Magnification of the (200) peaks in (*a*). (*c*,*d*) Elemental valence of the Fe and Co atoms in the BaM*_x_*Nb_(1−*x*)_O_3−*δ*_ nanocrystals, respectively.

Here, we report DMSs based on *M*-doped BaNbO_3−*δ*_, in which strong magnetism can be obtained at room temperature. The *M*-doped BaNbO_3−*δ*_ nanocrystals are prepared by the composite-hydroxide-mediated (CHM) method. The as-synthesized pure BaNbO_3−δ_ has a cubic lattice with *a* = *b* = *c* = 4.135 Å (figure 1*a,b*) and belongs to the P-m3m space group. This lattice parameter is larger than the theoretical value of 4.080 Å. Additionally, this lattice expansion is beneficial for accommodating more dopants. It can be proven by the XRD results with *M* doping that no second phase can be observed, as shown in figure 1*a*. The addition of Fe leads to a slight blue shift of diffraction peaks, indicating a decrease of the lattice parameter. While a red shift of diffraction peaks can be observed with the addition of Co, indicating an increase in lattice parameter, as shown in figure 1*b*. It is in accordance with the ionic radius of Nb^4+^ (68 pm), Fe^3+^ (64.5 pm) and Co^2+^ (74.5 pm). In addition, the emergence of a shoulder diffraction peak at 43.6° (figure 1*b*) with Co doping indicates a phase change from the cubic BaNbO_3_ to tetragonal BaCo*_x_*Nb_(1−*x*)_O_3−*δ*_. The change in phase structure of BaCo*_x_*Nb_(1−*x*)_O_3−*δ*_ powders indicates more Co atoms have been incorporated in the BaNbO_3_ matrix, implying better ferromagnetic properties than BaFe*_x_*Nb_(1−*x*)_O_3−*δ*_. Figure 1*c*,*d* shows the XPS results of BaM*_x_*Nb_(1−*x*)_O_3−*δ*_ nanocrystal, and the oxidation states of Fe and Co are +3 and +2, respectively, combining with the EDS mapping measurements (electronic supplementary material, figure S1), corroborating that the dopants are successfully doped in the corresponding compounds. Furthermore, the existence of oxygen defects in the as-synthesized samples by CHM methods is proven by the blueshift in the split binding energy of the O_1s states (electronic supplementary material, figure S2). The oxygen defects are another origin for the emergence of room temperature ferromagnetism in BaM*_x_*Nb_(1−*x*)_O_3−*δ*_ [18].

**Figure 2.**
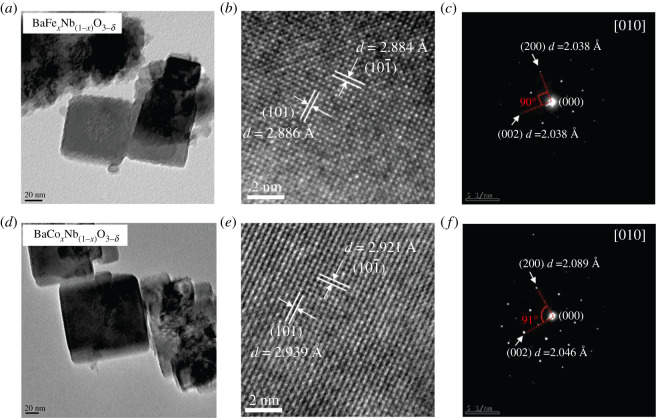
TEM characterizations of BaM*_x_*Nb_(1−*x*)_O_3−*δ*_ nanocrystals. (*a*,*d*) TEM images of Fe-, Co-doped the BaNbO_3−*δ*_ nanocrystals, respectively; (*b*,*e*), HRTEM images of Fe-, Co-doped the BaNbO_3−*δ*_ crystals, respectively; (*c*,*f*) corresponding SAED TEM images of samples.

To obtain further insight into the doped crystal lattices, the high-resolution transmission electron microscopy (HRTEM) and selection area electron diffraction transmission electron microscopy (SAED TEM) are used to characterize the crystalline structure of as-grown BaM*_x_*Nb_(1−*x*)_O_3−*δ*_ nanocrystals. As shown in figure 2*a–c*, the BaFe*_x_*Nb_(1−*x*)_O_3−*δ*_ nanocrystal has identical lattice spacings of orthorhombic (101)/(100) and (200)/(002) facets, proving that the Fe-doped BaNbO_3−δ_ maintains the cubic phase. However, the addition of Co increases the facet of (101) to 2.939 Å and, changes the BaCo*_x_*Nb_(1−*x*)_O_3−*δ*_ lattice to a tetragonal phase (figure 2*e*). It has a lattice spacing difference of 0.043 Å between (002) and (200) facets with an increased angle of approximately 91° (figure 2*f*), which matches well with the emergence of (002) peak in figure 1*b*.

## 4. Discussion

### 4.1. Room temperature ferromagnetism characterization

**Figure 3.**
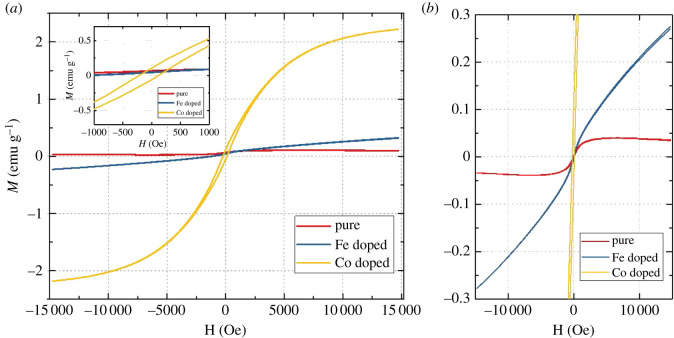
Magnetic properties of BaM*_x_*Nb_(1−*x*)_O_3−*δ*_. (*a*) Magnetic hysteresis curve *M*(*H*), as measured in an external field *H* of up to 15 kOe. The inset in the upper left corner shows the *M*_r_ and *H*_c_ of BaCo*_x_*Nb_(1−*x*)_O_3−*δ*_. (*b*) Magnified image shows the details of the *M*(*H*) curves of pure BaNbO_3−*δ*_ and BaFe*_x_*Nb_(1−*x*)_O_3−*δ*_.

To evaluate the ferromagnetism of BaM*_x_*Nb_(1−*x*)_O_3−*δ*_ nanocrystal, the field-dependent magnetization (*M*) was measured. Figure 3 shows the *M* versus applied magnetic field (*H*) curve with field sweeping from −15 000 to +15 000 Oe. As shown in figure 3*a*, the addition of *M* increases the ferromagnetism of BaMxNb_(1−*x*)_O_3−*δ*_ nanocrystal, especially for tetragonal BaCo*_x_*Nb_(1−*x*)_O_3−*δ*_, showing that an *M*_sat_ of 2.22 emu g^−1^ can be obtained at a driving field of approximately ±15 000 Oe, which is two orders of magnitude higher than that of pure BaNbO_3−*δ*_ (figure 3*b*). In addition, the BaFe*_x_*Nb_(1−*x*)_O_3−*δ*_ samples have an *M*_r_ of 0.084 emu g^−1^ and an *H*_c_ of 167.02 Oe. Although the addition of Co and Fe increases the magnetization of the samples, their hysteresis loops present an ‘S’ shape, indicating a long-range magnetic order [21].

### 4.2. Mechanism of room temperature ferromagnetism

**Figure 4.**
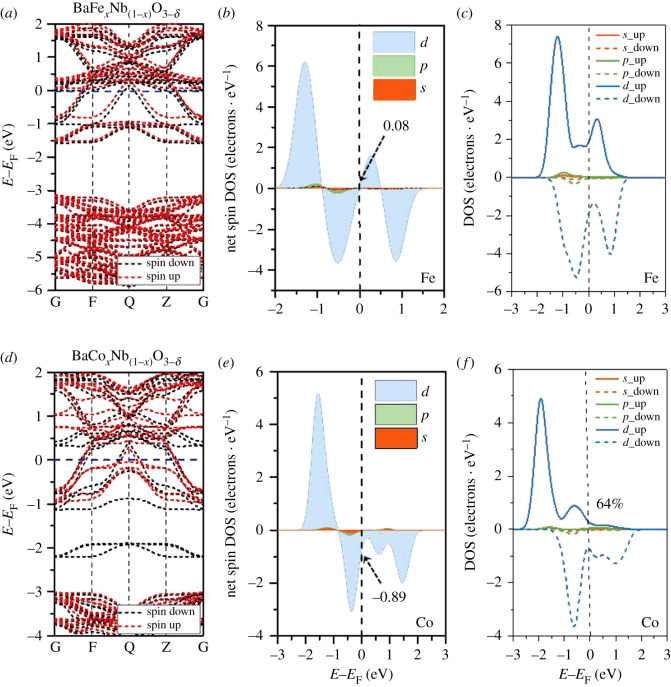
Calculated band structures and DOS of the doped atoms in the BaNbO_3−*δ*_ matrix. (*a*,*d*) Calculated band structures of Fe-, Co-doped BaNbO_3−*δ*_. (*b*,*e*) Calculated net spin PDOS of the Fe and Co atoms in the BaNbO_3−*δ*_ matrix, respectively. (*c*,*f*) Calculated spin-resolved PDOS of the Fe and Co atoms in the BaNbO_3−*δ*_ matrix, respectively.

To understand the physical mechanism of strong ferromagnetism maintained at room temperature, we investigated the electronic structure of the prepared materials based on *ab initio* methods. The 2 × 2 × 2 supercell crystal models were built with *M* elements replacing one of the Nb atoms, accompanied by one O vacancy in the oxygen octahedral cage, as shown in electronic supplementary material, figure S3. The calculated spin-resolved energy dispersion curves are shown in figure 4*a*,*d*. The carrier injection induced by doping transition metal elements and the existence of O defects lowers the conduction band minimum (CBM) below *E*_F_, presenting metallic n-type conduction. A small correlating spin splitting can be observed in the band structure of the BaFe*_x_*Nb_(1−*x*)_O_3−*δ*_ model (figure 4*a*), while the addition of Co leads to strong correlating spin splitting of more than approximately 1 eV (figure 4*d*); this result means that Co doping will promote stronger ferromagnetism. The doped *M* atom plays a dominant role in the spin polarization (see the supplemented partial density of states (DOS) of different atoms of BaM*_x_*Nb_(1−*x*)_O_3−*δ*_ in electronic supplementary material, figure S4), thereby presenting a local magnetic order. The calculated local net spin DOS of doped atoms at the *E*_F_ of BaCo*_x_*Nb_(1−*x*)_O_3−*δ*_ is more than one order of magnitude higher than BaFe*_x_*Nb_(1−*x*)_O_3−*δ*_, of which the net spin DOS has the maximum absolute value of approximately 0.89 electrons eV^−1^ (figure 4*b*,*e*). Thus, the predicted evolutionary trend remains in resolved DOS of doped atoms provides more details. Due to the weak correlating spin splitting of Fe, the major spin states almost overlap with the minor spin states, leading to a small spin polarization (figure 4*c*). The Co-doped BaNbO_3−δ_ shows the large spin splitting of approximately 1.26 eV, showing 64% spin polarization (figure 4*f*), which will contribute to maintaining strong ferromagnetism at room temperature.

**Figure 5.**
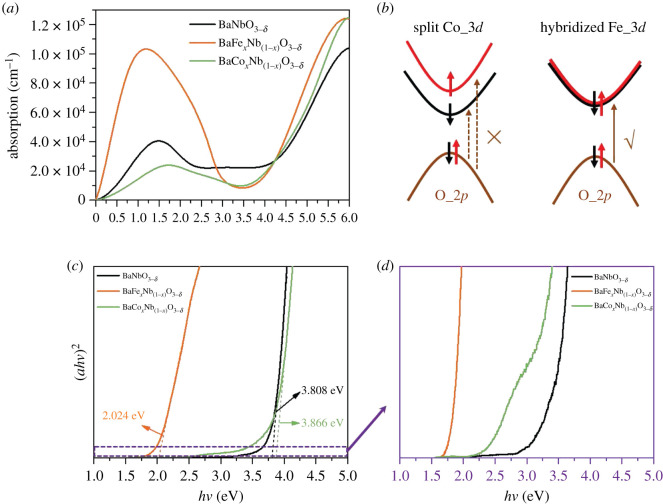
Absorption properties of BaM*_x_*Nb_(1−*x*)_O_3−*δ*_. (*a*) Calculated absorption coefficients of pure BaNbO_3−*δ*_, BaFe*_x_*Nb_(1−*x*)_O_3−*δ*_ and BaCo*_x_*Nb_(1−*x*)_O_3−*δ*_. (*b*) Schematic showing the mechanism of the trade-off effect between the magnetic and absorption properties in BaM*_x_*Nb_(1−*x*)_O_3−*δ*_. (*c*) UV–vis absorption spectra of the BaM*_x_*Nb_(1−*x*)_O_3−*δ*_ nanocrystals. (*d*) The magnified region showing the defect band absorptions of the BaM*_x_*Nb_(1−*x*)_O_3−*δ*_ nanocrystals.

Figure 5*a* shows the predicted optical absorption coefficient (*η*) of BaM*_x_*Nb_(1−*x*)_O_3−*δ*_ based on DFT calculations. The absorption induced by the transition between occupied O_2*p* orbitals and defect bands can be observed in all of the models due to doping transition metal elements and the existence of O defects. Co doping shows a small contribution to the defect band absorption (*hν* < 3.5 eV), which is six times smaller than the major absorption between the O_2*p* and Nb_4*d* orbitals (*hν* > 3.5 eV). However, BaFe*_x_*Nb_(1−*x*)_O_3−*δ*_ has a strong defect band absorption with a maximum *η* higher than 10^5^, which is comparable to the absorption between the O_2*p* and Nb_4*d* orbitals. The large differences in the defect band absorption between Co and Fe doping are ascribed to the strong local spin splitting of Co in the BaNbO_3−*δ*_ matrix, while the Fe in the BaNbO_3−*δ*_ matrix shows weak local spin splitting with strong hybridization of the opposite spin states near *E*_F_. As shown in the schematic of figure 5*b*, only states that have the same spin momentum are allowed, and the transition from degenerate O_2*p* orbitals to split Co_3*d* orbitals is forbidden. The above theoretical predictions are proven by the UV–vis absorption measurement results (spectra captured in a range of 1.55–6.22 eV). Figure 5*c* shows the absorption spectra of the prepared nanocrystal of BaM*_x_*Nb_(1−*x*)_O_3−*δ*_. Co-doped BaNbO_3−*δ*_ have dominant absorption band edges of 3.866, which show absorption properties similar to those of pure BaNbO_3−*δ*_. However, Fe-doped BaNbO_3−*δ*_ has absorption band edges located at 2.024 eV, indicating strong defect band transition absorption. The weak defect band absorptions of BaCo*_x_*Nb_(1−*x*)_O_3−*δ*_ and pure BaNbO_3−*δ*_ can be observed in figure 5*d*, and the results match well with the DFT calculations. The ferromagnetic performance and defect band absorption properties present a complementary relationship in BaM*_x_*Nb_(1−*x*)_O_3−*δ*_ DMSs, of which strong ferromagnetism corresponds to a relatively weak defect band absorption. The weak defect band absorption further verifies that the orbits near the Fermi level of BaCo*_x_*Nb_(1−*x*)_O_3−*δ*_ should have stronger Zeeman-type spin-polarization splitting.

## 5. Conclusion

Transition metal-doped BaNbO_3−*δ*_ nanocrystals are prepared by CHM methods. The as-prepared BaCo*_x_*Nb_(1−*x*)_O_3−*δ*_ nanocrystals present better ferromagnetic properties than BaFe*_x_*Nb_(1−*x*)_O_3−*δ*_. The strong ferromagnetism is obtained at room temperature with an *M*_sat_ of 2.22 emu g^−1^, *M*_r_ of 0.084 emu g^−1^ and *H*_c_ of 167.02 Oe, which is two orders of magnitude higher than that of pure BaNbO_3−*δ*_. The origin of the different ferromagnetic performances between Fe- and Co-doped BaNbO_3−*δ*_ nanocrystals is demonstrated by DFT calculations. Theoretical results show that the local net spin DOS of Fe and Co in the BaNbO_3−*δ*_ matrix are 0.08 and 0.89 electrons eV^−1^, respectively. Moreover, the strong spin polarization (64%) and high binding energy difference of the opposite spin states (approx. 1.26 eV) will contribute to maintaining the large *M*_sat_ of BaCo*_x_*Nb_(1−*x*)_O_3−*δ*_ at room temperature. The defective band transition properties of BaM*_x_*Nb_(1−*x*)_O_3−*δ*_ show a complementary relationship with their ferromagnetic performances since the band transition is forbidden between orbitals with different spin states, and this behaviour is corroborated by DFT calculations and absorption measurements. The tetragonal BaCo*_x_*Nb_(1−*x*)_O_3−*δ*_ within the morphology of bulk specimens provides a potential route for designing multifunctional spintronic devices that work at room temperature.

## Data accessibility

Our data are deposited at the Dryad Digital Repository: https://doi.org/10.5061/dryad.f4qrfj6wd [22].
